# Condition of the Bowels

**Published:** 1884-03

**Authors:** 


6. If a child, usually sprightly, holding itself up straight, is noticed to drop the head and seem languid and sleepy, or if it usually goes about from chair to chair, or is disposed to climb, but suddenly shows no disposition to do anything but lie down on the floor, there is something wrong in the stomach or bowels.

7. If there is crying and the legs are drawn up, there is indigestion, and the bowels are disordered.

8. In health, a child seldom carries the hands above the mouth ; if that is observed repeatedly, there is something wrong in the head ; the feet are cold, and they must be kept warm ; if the bowels have not acted within ten hours, give an enema or a teaspoonful of castor-oil every hour until there is an action.

9. A healthy child, especially if not over two years old, is often carrying the hand to the mouth ; but if it stops at the throat, croup is most likely forming ; notice instantly if the feet or hands are cold, and turn to the article on croup.

10. In the first months of infancy, if the little one is well, it nurses, plays awhile, and then falls into a gentle, easy, good sleep ; if it does not, if it is restless, especially if it starts up in its sleep, or wakes and whines, there is disturbance in the brain, and it should be seen to that the bowels are regular, feet warm, and food given at proper intervals, and of a suitable quality.

11. The first passages of an infant are dark colored, called the *meconium;* to bring this away is essential; if this is not done the child will suffer. But the first milk secreted, called the *colustrum,* acts as a purgative and carries the *meconium* before it; but if it does not come away oil must be given; sometimes warm water will answer; and in first confinements no milk appears sometimes for several days, hence any uneasiness of the first-born for the first few days may be caused by costiveness.

12. In health, young children go to sleep at once, and sleep quietly and soundly; if they are not well they do not lie down willingly at the regular hour for sleep, nor do they fall asleep at once, nor do they sleep continuously; there are frequent turnings and changings and wakings or startings up, often in alarm—then the bowels or head are out of order.

13. The passages of a healthy child are yellowish, and thicker than thick syrup, and are of uniform appearance, from three to four times a day ; less than two is costiveness. It should be rectified with an enema or castor oil. More than three or four, and as thin as milk and light colored, show diarrhea, and are rapidly debilitating. Keep the belly warm, especially the feet and hands. Do not feed at oftener than five hours' interval, and let the food be boiled rice, sago, tapioca, exclusively, with a little boiled milk, until there is a reduction in frequency, and greater consistency is manifested. If the stools are curdy, or green, or smell badly, or come out with considerable force, there is disease to be treated as just named.

